# Postoperative Complications of Flap Procedures in Chest Wall Defect Reconstruction: A Two-Center Experience

**DOI:** 10.3390/medicina60050834

**Published:** 2024-05-19

**Authors:** David Breidung, Sarina Delavari, Sebastian Grimme, Götz Habild, Moritz Billner, Dietmar Kraus, Bert Reichert, Ioannis-Fivos Megas

**Affiliations:** 1 Department of Plastic, Reconstructive and Hand Surgery, Center for Severe Burn Injuries, Klinikum Nürnberg, Paracelsus Medical University, 90471 Nuremberg, Germany; sarina.delavari@martha-maria.de (S.D.); sebastian.grimme@alumni.pmu.ac.at (S.G.); moritz.billner@klinikum-nuernberg.de (M.B.); bert.reichert@klinikum-nuernberg.de (B.R.); ioannis.megas@jsd.de (I.-F.M.); 2 Department of Health Management, Friedrich Alexander University Erlangen-Nuernberg (FAU), 91054 Erlangen, Germany; 3 Department of General and Visceral Surgery, Hospital Martha-Maria, 90491 Nuremberg, Germany; 4 Department of Orthopedic and Trauma Surgery, Center of Plastic Surgery, Hand Surgery and Microsurgery, Evangelisches Waldkrankenhaus Spandau, 13589 Berlin, Germany; 5 Department of General, Visceral and Thoracic Surgery, Klinikum Nürnberg, Paracelsus Medical University, 90419 Nuremberg, Germany; dietmar.kraus@klinikum-nuernberg.de

**Keywords:** reconstruction, chest wall, soft tissue reconstruction, free flap, pedicled flap

## Abstract

*Background and Objectives*: Chest wall defect reconstruction is a complex procedure aimed at restoring thoracic structural integrity after trauma, tumor removal, or congenital issues. In this study, postoperative complications were investigated to improve the care of patients with these critical conditions. *Materials and Methods*: A retrospective study of chest wall reconstructions from 2004 to 2023 was conducted at Klinikum Nürnberg and Evangelisches Waldkrankenhaus Spandau—Berlin. Data included patient demographics, comorbidities, defect etiology, surgery details, and complications using the Clavien–Dindo classification. *Results*: Among the 30 patients included in the study, a total of 35 complications occurred in 35 thoracic wall defect reconstructions. These complications were classified into 22 major and 13 minor cases. Major complications were more common in patients with cancer-related defects, and considerable variations were observed between free flap and pedicled flap surgeries. Notably, the use of the anterolateral thigh (ALT) flap with vastus lateralis muscle demonstrated promise, exhibiting fewer complications in select cases. The reconstruction of chest wall defects is associated with substantial complications regardless of the etiology of the defect and the particular surgical procedure used. Interestingly, there was a lower complication rate with free flap surgery than with pedicled flaps. *Conclusions*: The ALT flap with vastus lateralis muscle deserves further research in this field of reconstruction. Multidisciplinary approaches and informed patient discussions are crucial in this complex surgical field, emphasizing the need for ongoing research and technique refinement.

## 1. Introduction

Chest wall defect reconstruction is a complex surgical procedure to restore the structural integrity of the thoracic cavity after trauma, tumor resection, other thoracic or cardiac surgery, an infectious process, especially osteomyelitis of the sternum, or a congenital malformation. In the literature, there is a consensus among many authors that defects larger than 5 cm in diameter or those involving four or more ribs should be reconstructed [1,2,3,4]. This consensus arises from the recognition of the significant risk of complications such as lung herniation and respiratory compromise resulting from the paradoxical motion of the chest wall [3]. Surgeons widely acknowledge the need for reconstruction in these cases to restore the structural integrity of the chest wall and mitigate the associated risks to lung function. Reconstruction should enable the patient’s respiratory function, protect the viscera, and stabilize the shoulder girdle, making reconstruction critical to the patient’s quality of life and overall well-being [5]. Preoperatively, the patient’s general health and nutritional status should be optimized, and cardiac risk and pulmonary function should be analyzed, as reconstructions are generally associated with a postoperative ventilatory dysfunction [6,7].

Chest wall reconstructions often require a multidisciplinary approach due to the functional requirements, degree of difficulty, and specific anatomical considerations [8]. Local, regional, and free flaps are generally considered as options for the reconstruction of full-thickness chest wall defects [5]. The main applications for free flaps are when regional flaps cannot restore the defect due to their limited size or volume, when they are not available due to resection or damage (for example due to radiation), and when regional flaps are unable to cover the defect site due to distance or necessary rotation of the flap [5,9,10,11]. During reconstruction, emphasis should be placed on an airtight closure of the pleural cavity in order to reestablish the physiologic negative intrathoracic pressure for unhindered lung function [5,12]. Isaac et al. 2022 presented an algorithm for the reconstruction of chest wall defects that considered defect composition (soft tissue vs. soft tissue and bone defects, and a potential defect of the diaphragm), size, and characteristics such as a history of radiation or wound contamination as decision criteria [5].

The aim of this study was to identify and analyze the postoperative complications associated with chest wall reconstruction procedures and to investigate their implications for patient outcomes. By understanding these complications and their underlying risk factors, we can enhance our ability to provide optimal care and improve the overall success rates of chest wall defect reconstructions.

## 2. Materials and Methods

We performed a retrospective study of all cases of chest wall reconstruction with free or pedicled flap surgery in the Department of Plastic, Reconstructive and Hand Surgery, Center for Severe Burn Injuries at Klinikum Nürnberg and the Department of Orthopedic and Trauma Surgery, Center of Plastic Surgery, Hand Surgery and Microsurgery at Evangelisches Waldkrankenhaus Spandau—Berlin. The study was conducted in accordance with the Declaration of Helsinki. Ethics review and approval were waived for this study due to its retrospective design. Data were obtained from our hospital databases, and the study period was from January 2004 to June 2023. The inclusion criterion was the reconstruction of chest wall defects by free or pedicled flap surgery. No selection was made for age, comorbidities, or other case-characterizing factors. Cases were evaluated using discharge letters, operative reports, and admission forms. The operations on the patients were generally carried out by a multidisciplinary team of thoracic and plastic surgeons, with excision and mesh insertion being performed by thoracic surgeons and subsequent flap procedures by plastic surgeons. Over the included time frame of the study of almost 20 years, different surgeons participated in the surgeries, with the main surgeons of the cases being BR, DK, and GH.

Relevant data for this study were demographic data, comorbidities and risk factors, data on the etiology of the defects, previous possible radiation in the reconstruction or donor site, the size of the defect, information regarding the surgery, and postoperative complications. The scores of a visual analog scale (VAS) scoring system (0: no pain, 10: worst pain) documented by our nursing staff and attending doctors were used to evaluate postoperative pain. The Clavien–Dindo classification was used to classify postoperative complications [13]. The relevant complication severity grades for this study were Grades I–III. Surgical procedures were assigned to Grade III, whereas wound infections opened at the bedside were assigned to Grade I. Grade I and II complications were referred to as minor complications and Grade III complications as major complications in this study. Data were collected and analyzed using Excel^®^ version 16.85 (Microsoft, Redmond, WA, USA). Categorical variables were reported as totals. Percentages were not reported due to the small number of cases. Quantitative variables were reported as the mean with standard deviation or with minimum and maximum values, depending on the specific variable.

## 3. Results

A total of 30 patients were included in the study, 26 of whom were treated at Klinikum Nürnberg and four at Evangelisches Waldkrankenhaus Spandau—Berlin (see Table 1). Of these, 17 were men and 13 were women. The patients had an average age of 63.6 ± 10.1 years. Regarding the etiology of the defects, fourteen cases were due to cancer, seven cases were postoperative conditions, and nine cases were postoperative with established osteomyelitis. In the defect situations after cancer, the main types of tumors were sarcoma, (recurrent) breast cancer, and lung cancer. Postoperative defect situations included following heart valve replacement, coronary artery bypass surgery, coccidioidomycosis, and Boerhaave syndrome. The front of the thoracic wall was mainly affected in almost all defects.

Of the comorbidities and risk factors examined, cancer was the most common in the patient population, with 14 affected patients. This was followed by coronary artery disease and hypertension, each with ten affected patients. Seven patients had diabetes mellitus, six patients had had a history of myocardial infarction, and six patients suffered from heart failure. Four patients had a history of heart valve replacement, and three patients had chronic kidney disease. Less common concomitant diseases were pulmonary insufficiency and peripheral arterial disease with two patients each, and aortic stenosis, Boerhaave syndrome, history of pneumectomy, and history of pacemaker implantation with one affected patient each. Thirteen of the patients had undergone radiation in the past. Data on defect size were available in 20 patients, and the mean defect size was 146.2 ± 51.2 cm^2^. The smallest defect was 50 cm^2^, and the largest was 256 cm^2^.

**Figure 1:**
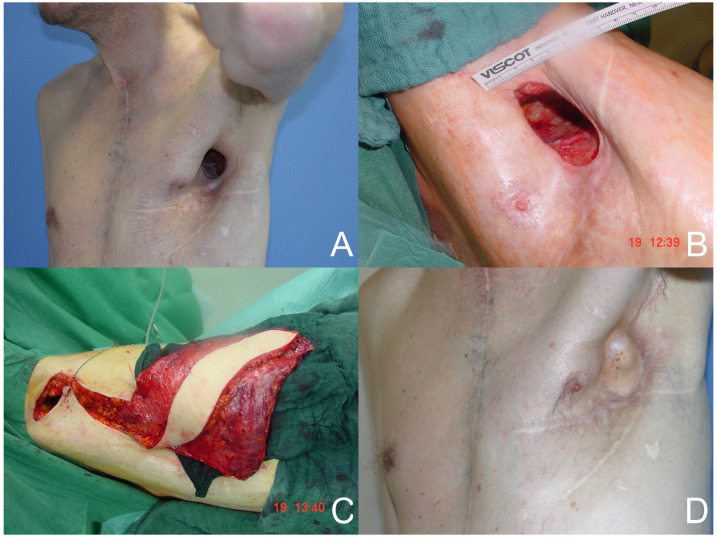
(**A**) Preoperative defect. (**B**) Intraoperative defect. (**C**) Free latissimus dorsi flap. (**D**) Postoperative result.

**Figure 2:**
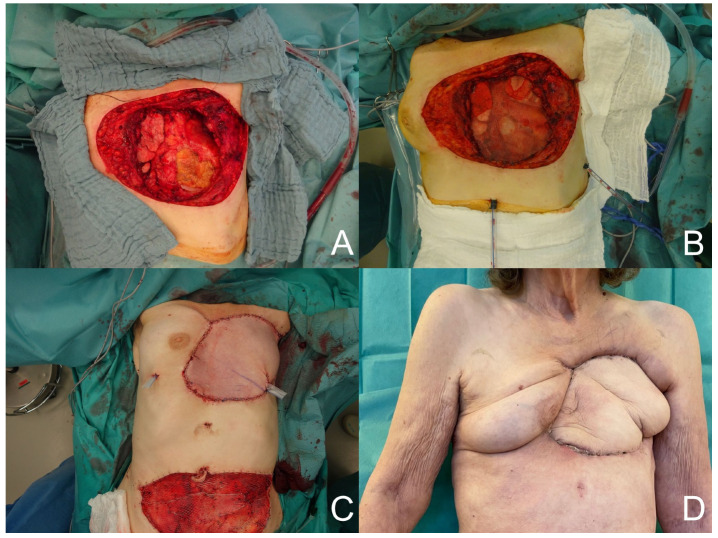
(**A**) Chest wall defect. (**B**) Mesh placement. (**C**) TRAM flap. (**D**) Postoperative result.

A total of 35 free or pedicled flap procedures were performed in these patients. Of these, nine were free flap procedures and twenty-six were pedicled flap procedures. Three free anterolateral thigh (ALT)-vastus lateralis flaps and one free ALT flap reconstruction were performed. In addition, five free latissimus dorsi flaps (see Figure 1) and thirteen pedicled latissimus dorsi flaps were performed. Other reconstructions with pedicled flaps included four vertical rectus abdominis (VRAM) flaps, one transverse rectus abdominis (TRAM) flap (see Figure 2), four pectoralis major flaps, one bilateral pectoralis major flap, one trapezius flap, one perforator-based rotation flap, and one anterior intercostal artery perforator (AICAP) flap. In the free flap procedures, the recipient artery was the internal thoracic artery in five cases, a branch of the axillary artery in three cases, and the circumflex scapular artery in one case. An end-to-end anastomosis was performed in seven cases, and an end-to-side anastomosis in two cases. In seven patients, the reconstructive surgeries involved the insertion of a type of surgical mesh. Seven patients were lost to follow-up. The duration of follow-up for the remaining patients was 2.6 ± 3.4 years.

A total of 35 complications occurred in the 35 thoracic wall defect reconstructions performed, of which 13 were minor complications and 22 were major complications. The most frequent complications were hematomas (seven cases) and partial necrosis (six cases). Complete necrosis occurred four times; wound healing complications, seroma, anemia and pneumonia occurred three times each. The formation of fistulas occurred in two cases. Reconstruction site infection, donor site infection, pleural effusion, and arm vein thrombosis each occurred once and were among the less common complications. Of the six partial necrosis cases, two were after a free latissimus dorsi procedure, two after VRAM flaps, and one each after a pedicled latissimus dorsi flap and a pedicled pectoralis flap. Two of the four full necroses occurred after defect reconstruction using pedicled latissimus dorsi flaps, one after a free latissimus dorsi flap procedure and one after an AICAP flap. A list of complications (classified according to the Clavien–Dindo classification) subdivided by reconstruction site, donor site, or other complications is presented in Table 2.

Complications were analyzed according to the etiology (see Table 3). There was a total of 17 postoperative complications in the reconstructions of defects resulting from cancer (14 cases). There were ten complications in postoperative defects (seven cases) and eight complications in postoperative defects with osteomyelitis (nine cases). Relatively as well as in absolute terms, the most major complications (12 out of 22) occurred in defect reconstructions due to cancer.

In Table 4, the parameters of the operative and postoperative course were subdivided according to the type of reconstructive procedure, free flap surgery or pedicled flap. The operating time for free flaps was on average more than two hours longer than for pedicled flaps. Maximum postoperative pain was higher after pedicled flaps on the first postoperative day and between the second and seventh postoperative day. A total of ten complications occurred after the nine free flap reconstructions, and twenty-two complications occurred after the twenty-six pedicled flap reconstructions. Grade II complications occurred relatively and absolutely more frequently after free flap surgery. Major complications were found to be relatively common after pedicled flap surgery. For the free flaps, these were four out of nine procedures compared with eighteen out of twenty-six procedures for pedicled flaps. Notably, the patients who underwent ALT + lateral vastus reconstruction had a relatively complication-free inpatient course, with only one complication (anemia requiring transfusion) occurring in the three affected patients. The duration of inpatient postoperative stay was relatively similar after free flap and pedicled flap procedures.

## 4. Discussion

This retrospective multicenter study outlines the challenging field of complex chest wall reconstruction. While the first approach to chest wall reconstruction with a pedicled latissimus dorsi flap was delineated by Purpura in 1908, the reconstructive surgeon’s toolbox has since evolved notably [14]. Despite a considerably high morbidity and mortality attributable to these complex operations, reconstruction has been shown to have a reasonable long-term survival while being safe and effective [9,15]. Many authors have put forth and adapted treatment algorithms for chest wall defects [5,12,16]. Apart from well-established pedicled flaps, microvascular free flap surgery has gained relevance and, despite higher complexity, longer operative time, and associated donor site morbidity, may be superior to regional myocutaneous flap reconstruction in selected circumstances such as following radiotherapy for extensive defects or when local options have been exhausted [9,11,12,15,17].

At first glance, our data may suggest an anomalously high complication rate, regardless of the specific surgery technique. In 35 procedures on 30 patients, a total of 35 complications occurred, resulting in a relative overall complication rate of 100% (37% major and 63% minor). Contrasting this and having used mainly pedicled latissimus dorsi flaps, Groth et al. reported an overall complication rate of 50% (12.5% major and 37.5% minor complications) in a more than decade younger collective of 32 patients with mainly oncologic reconstructions [15]. In their definition of major complications, compared to our results, only complications requiring surgical procedures were considered. Hereby, minor complications were not further classified. They reported two partial but no full necrosis, which is a considerable difference to our data, where six partial and four full necrosis occurred in the entire population. Generally, in advance of contextualizing complication rates with the existing literature regarding chest wall reconstruction, a sufficiently informative comparison is a challenging task, since a catalog of possible primary diseases and patient-dependent factors would make a case-comparison impossible. There may be different explanatory theories. First of all, it is reasonable to report higher complication rates in patients with different primary diseases with complex multidisciplinary aspects such as Groth and colleagues, who included mainly patients with local breast cancer. Second, their analysis deferred to mainly soft tissue reconstruction (81.25%) and only four thoracotomies (i.e., full-thickness chest wall reconstructions were performed in their collective). Third, their study lacked a report of the defect sizes, which is a known predicting factor for outcome [18]. Beyond that, the additionally reported complications differed significantly: no cases of hematoma, pneumonia, or anemia were reported, which are relatively common anticipated complications after such operations [12,18]. In contrast to our study, wound dehiscence was considerably more common and was the most frequent complication in the study by Groth et al., occurring in 22% of patients. However, cases with wound dehiscence never required revision and thus categorized as minor complications. On the other hand, their seroma rate of 9.3% matched our data.

In comparison, Corkum et al. (2020) found an overall complication rate of 61% after chest wall reconstruction in their study, with 59 patients and a mean age of 53 years [18]. Regarding flap necrosis, they reported three cases among the twenty-four pedicled muscle flaps and four free flaps performed. According to Clavien–Dindo, their minor and major complication rates were 37.3% and 39%, respectively. Their reported major complication frequency was comparable to our data.

Losken et al. (2004) proposed a treatment algorithm having analyzed 158 patients undergoing full-thickness thoracic wall reconstruction with mesh prosthesis and subsequent primary closure, pedicled flap, or free flap coverage [12]. Their collective had comparable demographics, however, only 15% of patients received radiotherapy, plus sternum osteomyelitis cases were excluded; hence their low complication rate for reconstruction of 27%, whereby pneumonia (15%) represented the most common complication. Additionally, they postulated radiotherapy and hypertension as possible risk factors.

Sauerbier et al. (2011) analyzed 69 cases of thoracic wall reconstruction for tumor, of which 30.4% were microsurgical [17]. They reported three total flap losses, and finally, an unspecified complication rate of 44.6%, which approximately matches our percentage of major complications. Our analysis suggests that the pedicled flap subgroup may tend to have higher rates of complications compared to the free flap subgroup. However, it is important to recognize the inherent limitations of our retrospective study design in this regard. Nonetheless, our results mark an interesting avenue for further investigation, and future studies with more comprehensive data collection methods are warranted. Potentially, the fear of the complexity of vascular microsurgery or simply health economic boundaries involved in free flap reconstruction leads to the surgeon’s preference for local, and thus more straightforward approaches for thoracic wall reconstruction. However, in these borderline cases, if free flap surgery is properly and thoroughly planned and executed, could yield superior outcomes. Contradicting common beliefs, it is clearly depicted by our results that free flap surgery, if performed by trained surgeons, is potentially safer than opting for local flap options at any cost. Nevertheless, it should be noted in this context that the pedicle-based latissimus dorsi flap is still a reliable reconstructive method in the absence of additional complicating factors and should be considered the workhorse for larger full-thickness thoracic defects [15,19].

Furthermore, besides being rarely appreciated in the literature, in our collective, ALT with vastus lateralis muscle for chest wall reconstruction stood out with only one Grade II complication (anemia requiring transfusion) after three performed surgical procedures. In 2010, Di Candia and his institute reported a major-complication-free course of five cases of chest wall reconstruction with ALT flaps, in some cases involving portions of the vastus lateralis [20]. Despite the variable anatomy and possible musculocutaneous perforators as outlined by Song, the considerably long transplant plus the favorable esthetic result in typically thinner oncologic patients are mentionable advantages [21]. Moreover, increased anatomical distance to irradiated or potentially compromised tissue locoregional to the thoracic defect seam are plausible advantages of the ALT. Consequently, further research, particularly regarding ALT with the vastus lateralis muscle for chest wall reconstruction, would be of great interest.

In summary, we can postulate key points to evaluate before selecting the appropriate soft-tissue reconstruction method of thoracic wall defects:

1. Interdisciplinary assessment of the underlying disease, patient morbidity, prognosis, and functional demand.
2. Interdisciplinary treatment plan for the underlying disease (curative vs. palliative).
3. Structural support of the rib cage if more than two ribs are absent or the chest wall biomechanics are compromised.
4. Evaluate local options if no history of radiotherapy or possible local extension of disease.
5. Consider economic and public health aspect of complex free flap surgery.
6. Stages of surgery needed.
7. Assessing esthetic appeal and donor site morbidity.

Our study possesses several notable strengths. First, it boasts a lengthy duration, spanning from 2004 to 2023. This extensive timeframe and the multicenter study design allowed us to draw from a substantial pool of cases, enhancing the real-world relevance of our findings. Furthermore, we included a diverse patient population comprising individuals with a range of medical conditions who underwent various surgical approaches. This diversity adds to the applicability and robustness of our results. We also conducted meticulous data collection, gathering information on patient demographics, comorbidities, and complications. The use of the Clavien–Dindo classification system ensures consistency in our assessment of complications and provides valuable contexualization of our results against the background of the existing literature.

However, our study also has its limitations. Being retrospective in nature, it may be subject to selection bias, and establishing causality can be challenging. We gathered data from two medical centers, which may have introduced variations in patient populations and practices, potentially affecting the generalizability of our findings. While comprehensive across our participating institutions, the sample size, though substantial, might not have been sufficient to detect rare complications or draw detailed conclusions for specific subgroups at each center. In order to achieve a high number of cases and to reduce bias due to possible selection criteria, we did not exclude any further cases from the study. Variability in the documentation of comorbidities and surgical details across centers could affect the comprehensiveness of our analysis. To address this, we adapted the data collection to involve multiple sources including discharge letters, surgical reports, and admission forms and conducted these independently by two evaluators. Additionally, changes in surgical techniques and practices over the study period may have influenced the outcomes and introduced challenges in making comparisons.

It should be noted that we focused primarily on the short-term complications in this study, while we deliberately neglected the long-term outcomes, and thus the patients’ quality of life. This was because long-term quality of life can be affected by numerous variables of non-standardized postoperative care and the motivation of each individual patient, rehabilitation, or disease progression. To minimize potential bias in relation to complications, we used an established classification system to standardize our assessment of complications and allow for a comparison with the literature. To our knowledge, there are no data on the long-term outcomes and quality of life following the flap reconstruction of thoracic defects, which would display an interesting yet different questioning, possibly respecting donor-site morbidity and functioning in activities of daily living to a greater degree. Moreover, the complications reported in our sample were thoroughly classified and listed, regardless of a surgery-specific etiology or else. Elucidating our data, the complication rate must acknowledge the thoroughness of both planning surgery on critically ill patients and detecting every demarking complication appropriately. Despite the limitations, our multicenter study provides valuable insights into the complexities of chest wall reconstruction. It serves as a new puzzle stone for future research on the scope of microsurgical techniques and clinical advancements in this challenging and interdisciplinary field.

## 5. Conclusions

In summary, our retrospective multicenter study on chest wall defect reconstruction highlights that these surgeries are associated with significant complications. It underscores the importance of comprehensive discussions with patients before proceeding with these procedures to ensure that they are well-informed about the potential risks and outcomes. While there appeared to be a trend suggesting lower rates of major complications with free flap surgery compared to pedicled flaps, further investigation is warranted to confirm these observations and to account for potential confounding variables that may influence the outcomes. Additionally, our findings suggest that the use of the anterolateral thigh flap with the vastus lateralis muscle holds promise as a viable option for select cases. Nonetheless, additional studies are required to validate its potential. Chest wall defect reconstruction is a complex surgical undertaking with inherent challenges. To optimize patient outcomes, thorough preoperative discussions and a multidisciplinary approach are vital. This study emphasizes the ongoing need for research to refine surgical techniques and enhance patient care in this demanding field.

## Figures and Tables

**Table 1:** Cases of thoracic wall reconstruction with free or pedicled flap surgery.

Age	Sex	Etiology	Comorbidities and Risk Factors	Radiation	Size of Defect (cm × cm)	Type	Flap	Mesh	Recipient Artery	Anastomosis
73	f	Surgery with osteomyelitis	MI, CAD, CKD	No	16 × 7	Free	ALT-vastus lateralis		Internal thoracic artery	ES
76	m	Surgery with osteomyelitis	DM, Obesity, HF, AS, CAD	No	23 × 7	Free	ALT-vastus lateralis		Internal thoracic artery	EE
67	m	Surgery with osteomyelitis	DM, Obesity, MI, CAD, HTN, PAD	No	18 × 8	Free	ALT-vastus lateralis		Internal thoracic artery	EE
41	m	Cancer	Cancer	Yes		Free	ALT		Internal thoracic artery	EE
45	f	Cancer	Cancer	Yes	20 × 5	Free	Latissimus dorsi	Vicryl-Prolene-Composite	Axillary arterybranch	ES
59	m	Cancer	Cancer	Yes		Free	Latissimus dorsi		Circumflex scapular artery	EE
59	m	Cancer	Cancer	Yes		Free	Latissimus dorsi		Axillary artery branch	EE
42	m	Surgery	Pneumoectomy	No		Free	Latissimus dorsi		Axillary arterybranch	EE
67	f	Cancer	Cancer	Yes	16 × 16	Free	Latissimus dorsi		Internal thoracic artery	EE
						Pedicled	VRAM			
65	f	Surgery	Obesity, MI, HF, VR, HTN, PAD	No	20 × 9	Pedicled	Latissimus dorsi			
69	f	Cancer	Cancer	Yes		Pedicled	Latissimus dorsi	Vicryl-Prolene-Composite		
71	m	Cancer	Cancer, Obesity, PI, HTN	Yes	15 × 15	Pedicled	Latissimus dorsi	Polypropylene		
59	m	Surgery with osteomyelitis	DM, Obesity, CAD, HTN	No		Pedicled	Latissimus dorsi			
58	m	Surgery	DM, Obesity, CAD, VR, AB	No	22 × 8	Pedicled	Latissimus dorsi			
						Pedicled	Latissimus dorsi			
57	f	Surgery with osteomyelitis	CAD	No	21 × 9	Pedicled	Latissimus dorsi			
						Pedicled	Latissimus dorsi			
50	f	Cancer	Cancer	Yes		Pedicled	Latissimus dorsi			
78	f	Cancer	Cancer	Yes	16 × 13	Pedicled	Latissimus dorsi			
78	f	Cancer	Cancer	Yes	15 × 10	Pedicled	Latissimus dorsi	Monocryl-Prolene-Composite		
68	m	Surgery	Boerhaave syndrome	No	10 × 5	Pedicled	Latissimus dorsi			
67	m	Surgery with osteomyelitis	HF	No	11 × 6.5	Pedicled	Latissimus dorsi			
						Pedicled	Pectoralis major			
60	m	Surgery	Obesity, CAD, PI, HTN	No	16 × 7	Pedicled	Pectoralis major			
63	m	Surgery with osteomyelitis	DM, MI, HTN	No	12 × 6	Pedicled	Pectoralis major			
76	f	Cancer	Cancer, MI, PPM, CRD	Yes	15 × 10	Pedicled	Pectoralis major	Polypropylene		
69	m	Surgery	DM, HF, CAD, HTN	No	20 × 7	Pedicled	Bilateral Pectoralis major			
75	m	Surgery	HF, CAD, VR, CKD, HTN	No		Pedicled	VRAM			
69	f	Surgery with osteomyelitis	DM, MI, CAD, CKD HTN	No		Pedicled	VRAM			
71	f	Cancer	Cancer	Yes	12 × 10	Pedicled	TRAM	Polypropylene		
68	m	Cancer	Cancer	Yes		Pedicled	Trapezius			
52	m	Cancer	HF, VR	No	13 × 4	Pedicled	AICAP			
						Pedicled	VRAM			
57	f	Cancer	Cancer	No	8 × 8	Pedicled	Rotation flap	Polypropylene		

AB: alcohol abuse, AICAP: anterior intercostal artery perforator, ALT: anterolateral thigh, AS: aortic stenosis, CAD: coronary artery disease, CKD: chronic kidney disease, DM: diabetes mellitus, EE: end-to-end, ES: end-to-side, f: female, HF: heart failure, HTN: hypertension, m: male, MI: status post myocardial infarction, PAD: Peripheral artery disease, PI: pulmonary insufficiency, PPM: status post pacemaker implantation, TRAM: transverse rectus abdominis, VR: status post valve replacement, VRAM: vertical rectus abdominis.

**Table 2:** Postoperative complications classified according to the Clavien–Dindo classification.

Complications		Grade I		Grade II		Grade III	
		N	%	N	%	N	%
Reconstructive Site	Infection					1	100
	Fistula			1	50	1	50
	Hematoma					7	100
	Wound healing complications			1	50	1	50
	Partial necrosis					6	100
	Full necrosis					4	100
Donor Site	Infection					1	100
	Wound healing complications			1	100		
	Seroma	3	100				
Other complications	Anemia			3	100		
	Pneumonia			3	100		
	Pleural effusion					1	100
	Arm vein thrombosis			1	100		
Total		3		10		22	

**Table 3:** Postoperative complications classified according to the Clavien–Dindo classification subdivided by the etiology of the defect.

Etiology	Grade I	Grade II	Grade III
Cancer (*n* = 14)	1	4	12
Surgery (*n* = 7)	1	5	4
Surgery with osteomyelitis (*n* = 9)	1	1	6

**Table 4:** Parameters of the operative and postoperative course subdivided by the type of reconstructive surgery.

Parameter	Free Flap (n = 9)	Pedicled Flap (n = 26)
Operating time, minutes	370.2 ± 123.3	232.9 ± 113.4
Maximum pain first postoperative day ^a^	4 ± 1.7	4.5 ± 1.6
Maximum pain postoperative days 2–7 ^a^	3.7 ± 1.2	5.2 ± 1.7
Postoperative complications ^b^		
Grade I		3
Grade II	6	4
Grade III	4	18
Partial necrosis	2	4
Full necrosis	1	3
Postoperative hospital stay, days	24.0 ± 17.6	23.4 ± 13.2

^a^ Using a visual analog scale (VAS). ^b^ Classified according to the Clavien–Dindo classification.

## Data Availability

Data are contained within the article.
